# Energetic Oxygen and Sulfur Charge States in the Outer Jovian Magnetosphere: Insights From the Cassini Jupiter Flyby

**DOI:** 10.1029/2019GL085185

**Published:** 2019-11-08

**Authors:** R. C. Allen, C. P. Paranicas, F. Bagenal, S. K. Vines, D. C. Hamilton, F. Allegrini, G. Clark, P. A. Delamere, T. K. Kim, S. M. Krimigis, D. G. Mitchell, T. H. Smith, R. J. Wilson

**Affiliations:** ^1^ Applied Physics Laboratory Johns Hopkins University Laurel MD USA; ^2^ Laboratory for Atmospheric and Space Physics University of Colorado Boulder Boulder CO USA; ^3^ Department of Physics University of Maryland College Park MD USA; ^4^ Space Science and Engineering Division Southwest Research Institute San Antonio TX USA; ^5^ Department of Physics and Astronomy University of Texas at San Antonio San Antonio TX USA; ^6^ Geophysical Institute University of Alaska Fairbanks Fairbanks AK USA

**Keywords:** Jupiter, Composition, Cassini, Juno

## Abstract

On 10 January 2001, Cassini briefly entered into the magnetosphere of Jupiter, en route to Saturn. During this excursion into the Jovian magnetosphere, the Cassini Magnetosphere Imaging Instrument/Charge‐Energy‐Mass Spectrometer detected oxygen and sulfur ions. While Charge‐Energy‐Mass Spectrometer can distinguish between oxygen and sulfur charge states directly, only 95.9 ± 2.9 keV/*e* ions were sampled during this interval, allowing for a long time integration of the tenuous outer magnetospheric (~200 R_J_) plasma at one energy. For this brief interval for the 95.9 keV/*e* ions, 96% of oxygen ions were O^+^, with the other 4% as O^2+^, while 25% of the energetic sulfur ions were S^+^, 42% S^2+^, and 33% S^3+^. The S^2+^/O^+^ flux ratio was observed to be 0.35 (±0.06 Poisson error).

## Key Points

- Cassini measured the relative charge state abundances of 95.9 keV/*e* oxygen and sulfur in the outer magnetosphere (~200 R_J_) of Jupiter
- The flux of 95.9 keV/*e* O^+^ was higher than that of S^2+^, with S^2+^ being the most abundant charge state among sulfur ions
- The relative abundances of 95.9 keV/*e* heavy ions are compared to thermal ions in the inner to middle magnetosphere

## 1 Introduction

Galilean moons provide the majority of the plasma within the magnetosphere of Jupiter (see reviews by Krupp et al., 2004; Thomas et al., 2004). Atmospheric loss from the moon Io contributes about a ton per second of neutral material to the magnetosphere, most of which is SO_2_ and dissociated products (e.g., Thomas et al., 2004). Additionally, Europa has been reported as a minor source (in comparison to Io) of atomic oxygen and H_2_ through sputtering and atmospheric loss (e.g., Hall et al., 1995; Hansen et al., 2005; Mauk et al., 2003). These neutral populations, from both Io and Europa, can become ionized through electron impact and charge exchange (see Thomas et al., 2004). Upon ionization, the ions are picked up into the corotating magnetosphere with energies of ~0.5 keV/nuc of the order of the difference between the local Keplerian and corotational kinetic energies. Plasma corotating in the magnetosphere will also experience various dynamical processes, such as interchange, in which flux tubes with cooler denser plasma move radially outward, while flux tubes with more tenuous hotter plasma move planetward (see summary in Krupp et al., 2004).

While the distribution and abundances of ions at Jupiter have been determined from a variety of measurements, very little is known about the distribution of heavy ion charge state composition. During the Voyager 1 flyby of Jupiter, the Voyager Plasma Science (PLS) instrument studied the flux of different m/q groups of 0.01–5.95 keV/*e* ions finding the magnetosphere is dominated by ions with m/q of 16 amu/*e*, which comprised of both O^+^ and S^2+^ (Bagenal & Sullivan, 1981; McNutt et al., 1981). At high energies (0.60–1.15 MeV/nuc), observations from the Voyager Low‐Energy Particle Telescope, which resolves the mass but not charge state of ions, found the S/O ratio generally decreases with radial distance from Jupiter until leveling off near ~30 R_J_, reaching outer magnetospheric (out to ~170 R_J_) values between ~10% and 40% (Hamilton et al., 1981). A combination of both Voyager Low Energy Charged Particle and Galileo Energetic Particles Detector (EPD) measurements found the energetic (few tens of keV to tens of MeV) oxygen ion abundance was higher near Europa than near Io. This difference in abundance was suggested to be due to the higher charge exchange cross sections for O^n+^ interacting with neutral O than for S^n+^, mainly affecting ions in the higher neutral density inner magnetosphere (Mauk et al., 2004; Paranicas et al., 2003). Nénon and André (2019) additionally reported S^n+^ loss due to charge exchange with the Europa neutral tori using Galileo EPD, but didn't investigate O^n+^. Crary et al. (1998) documented the heavy ion charge states of O and S using PLS data to estimate the relative 8.6 eV/*e* to 53 keV/*e* ion abundance inside the Io torus and reported higher oxygen abundances than observed by the Voyager flybys. Galileo Heavy Ion Counter Low Energy Telecone B observations of energetic particles during Io encounters, accompanied by a Monte Carlo simulation, suggested MeV/nuc range O and S were nearly fully stripped, unlike the bulk population (Selesnick & Cohen, 2009). During the Ulysses flyby, the Solar Wind Ion Composition Spectrometer, which resolved the 0.6–60 keV ion composition, sampled O^+^ and S^2+^ in the Jovian magnetosphere, but only count rates were reported rather than instrument‐corrected fluxes (Galvin et al., 1993; Geiss et al., 1992). Recent analysis of the Juno Jovian Auroral Distributions Experiment‐Ion (JADE‐I) sensor data has incorporated detailed forward modeling to discriminate between O^+^ and S^2+^ (Kim et al., 2019). Additionally, remote sensing has been utilized to infer plasma charge states, such as with the Cassini Ultraviolet Imaging Spectrograph (UVIS) subsystem (Steffl, Bagenal, et al., 2004; Steffl, Stewart, et al., 2004; Steffl et al., 2006), Hisaki extreme ultraviolet (EUV; Yoshioka et al., 2017), and the Chandra X‐ray Observatory (Elsner et al., 2002).

To fill in the gaps in previous measurements and better understand the underlying physics, physical chemistry models have also been developed to derive the composition based on reactions from neutral S and O evolving over a set transport time period (e.g., Delamere et al., 2005). Recent reanalysis of the Voyager PLS data has improved upon the original analysis through using the Delamere et al. (2005) physical chemistry model to constrain the O^+^/S^2+^ (Bagenal et al., 2017; Bodisch et al., 2017; Dougherty et al., 2017). However, the limited number of observations directly distinguishing between S^2+^ and O^+^ still leads to ambiguity, and so additional observations, particularly in different regions of the magnetosphere over different energy ranges, are needed to better constrain models and understanding.

The Magnetosphere Imaging Instrument/Charge‐Energy‐Mass Spectrometer (MIMI/CHEMS) sensor onboard Cassini was able to separate keV‐range ions by their mass and charge state using triple coincidence measurements. During its distant Jupiter flyby in 2001, observations from the plasma wave instrument indicated Cassini flew into the distant Jovian magnetosphere (Kurth et al., 2002) during an unusual expansion of the outer magnetosphere due to low solar wind dynamic pressure. CHEMS measured ions for approximately 1 hr inside Jupiter's magnetosphere but only at one energy. In this paper, we present an analysis of the CHEMS observations and describe the relative flux content of the various heavy ion charge states. We compare our findings to the small data set of energetic ion charge states previously obtained at Jupiter and place our results into the context of the system.

## 2 Methodology

The Cassini spacecraft launched on 15 October 1997 executed a gravity assist flyby of Jupiter in December 2000–January 2001. Cassini only made two brief excursions into the magnetosphere of Jupiter (9 January 2001 12:50–20:35 UT and 10 January 2001 06:55–20:35 UT) near 200 R_J_ from Jupiter due to unusually low solar wind dynamic pressure allowing for an inflated magnetosphere (Kurth et al., 2002). In this study, we use data from MIMI/CHEMS (Krimigis et al., 2004). CHEMS provides triple coincidence ion measurements through the combined use of an electrostatic analyzer (ESA), time‐of‐flight (ToF) chamber, and solid‐state detector (SSD), allowing for unambiguous measurements of ion species' mass and charge states. In particular, CHEMS can discriminate between O^+^ and S^2+^ (both having a m/q of 16 amu/*e*) unlike observations from standard ESA‐ToF and ToF‐SSD systems.

**Figure 1:**
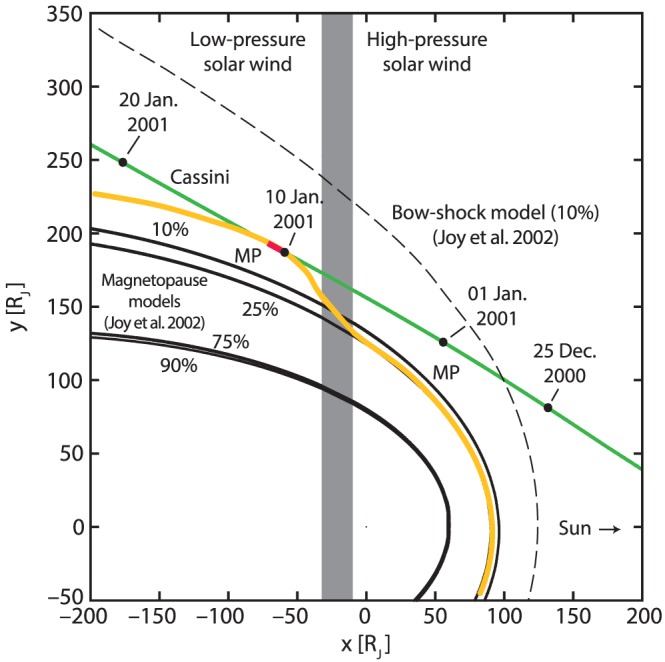
During the 10 January 2001 magnetospheric excursion, a solar wind pressure increase was passing over the magnetosphere of Jupiter but had yet to reach Cassini (see Kurth et al., 2002). The yellow line denotes an estimated magnetopause, while the red segment denotes the time Cassini was within the Jovian magnetosphere. Solid (dashed) black curves show modeled magnetopause (bow shock) boundaries using the Joy et al. (2002) models. The gray box shows the approximate region of the solar wind pressure enhancement moving through the system at the time Cassini was within the magnetosphere. Adapted from Kurth et al. (2002).

The Cassini Jupiter flyby trajectory is shown by the green curve in Figure 1, with the red segment showing the region where Cassini was within the magnetosphere of Jupiter on 10 January 2001. For the interval of interest, Cassini is located at a radial distance of ~200 R_J_, a local time of ~1915 hr, and at low (<10°) absolute magnetic latitudes (see Kurth et al., 2002).

On 7 January 2001, the data processing unit for MIMI shut down before coming back online on 10 January 2001. For this reason, there are no CHEMS observations for the 9 January 2001 magnetospheric interval. Due to the start‐up sequence of CHEMS, the instrument became functional again during the 10 January 2001 magnetosphere excursion, but with a fixed ESA voltage, limiting observations to a single energy (95.9 ± 2.9 keV/*e*) for the last 79 min of the magnetospheric interval (19:16 to 20:35 UT). The fixed ESA voltage allowed for a long accumulation of energetic magnetospheric ion species observations using triple coincidence measurements. This paper shows flux ratios of ion charge states from flux reported in units of [cm^2^·s^‐1^·sr^‐1^·(keV/*e*)^‐1^]. For this reason, caution must be applied when comparing the results to density or flux ratios using a noncharge‐dependent flux unit.

During this interval, the microchannel plates (MCP) were still increasing in voltage due to the start‐up procedure. The relative counts between oxygen and sulfur charge states didn't significantly change during these steps (not shown), suggesting the MCP voltage steps weren't significantly altering the efficiencies of O^+^ and S^2+^ relative to each other. However, these steps would have resulted in overall lower efficiencies earlier in the interval when the MCP voltage was lower. For this reason, this study refrains from reporting absolute flux values, and instead reports results as ratios, which are unaffected by these changes in MCP voltage.

## 3 Results

**Figure 2:**
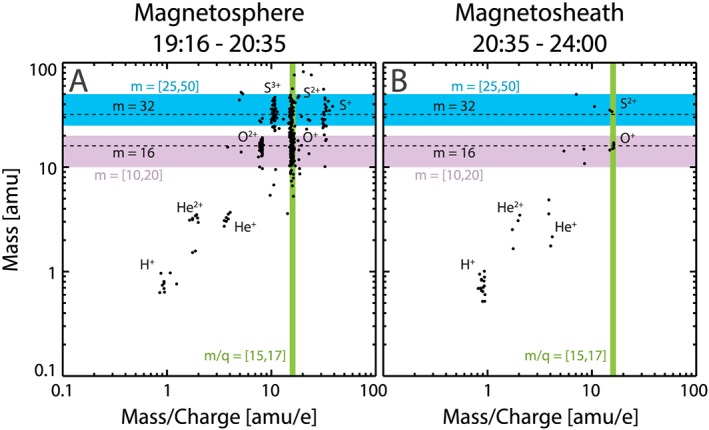
Pulse height analysis counts for (A) the magnetospheric excursion and (B) the magnetosheath after exiting the magnetosphere on 10 January 2001. The green vertical bar shows the mass‐per‐charge range of O^+^ and S^2+^ populations used in Figure 3 (mass per charge = [15,17] amu/*e*), while the blue and purple horizontal bars show the mass ranges used in Figure 4 for sulfur (m = [25,50] amu) and oxygen (m = [10,20] amu), respectfully. Horizontal dashed lines denote mass 16 (oxygen) and 32 amu (sulfur).

**Figure 3:**
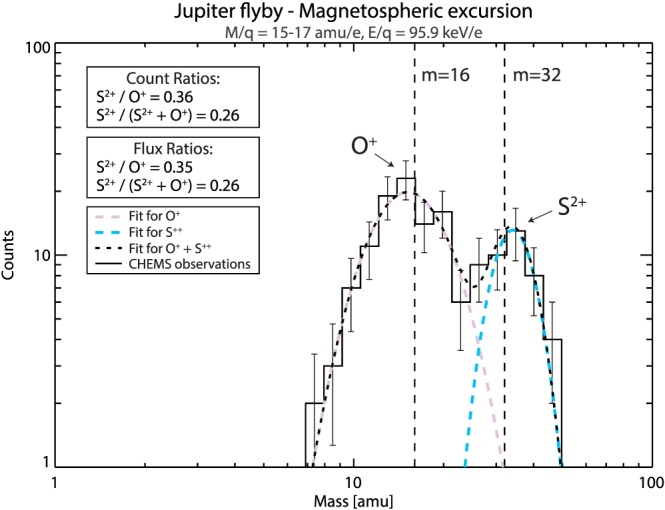
Histogram of counts in the mass‐per‐charge range of 15–17 amu/*e* with Poisson error. The purple (blue) dashed curves show the weighted Gaussian fit of the histogram for O^+^ (S^2+^). The black dashed curve shows the sum of the two fits.

Raw pulse height analysis (PHA) counts ordered by mass versus m/q are shown in Figure 2 with Panel A (B) showing only measurements taken within (outside of) the magnetosphere on 10 January 2001. Both oxygen and sulfur ions are detected within the magnetosphere (Figure 2A). While oxygen and sulfur were detected in the magnetosheath, these abundances are far lower and may be a result of magnetospheric ions escaping into the magnetosheath (see Krimigis et al., 2002; Krupp et al., 2002, 2004; Mauk et al., 2019). Solar wind‐originating populations (i.e., He^2+^) are observed within the magnetospheric interval, which have been previously detected in planetary magnetospheres (e.g., Allen, Livi, & Goldstein, 2016; Allen, Livi, Vines, et al., 2016; Allen et al., 2017, 2018; Ipavich & Scholer, 1983). The green box in Figure 2 denotes the mass‐per‐charge (m/q) range of 15–17 amu/*e*, in which both O^+^ and S^2+^ are observed. All of the magnetospheric interval PHA counts from this m/q range are plotted as a histogram of counts versus mass, with Poisson error bars, in Figure 3. The purple and blue dashed curves denote weighted Gaussian fits of the O^+^ and S^2+^ peaks, respectively, while the black dashed curve shows the sum of both fits. There are two distinct peaks in Figure 3, corresponding to O^+^ (16 amu) and S^2+^ (32 amu). The dashed black curve is seen to agree very well with the observations from CHEMS. From this measurement, we can reliably estimate the number of counts of both species and compute flux ratios for this energy, yielding an S^2+^/O^+^ flux ratio of 0.35 (±0.06 Poisson error).

**Figure 4:**
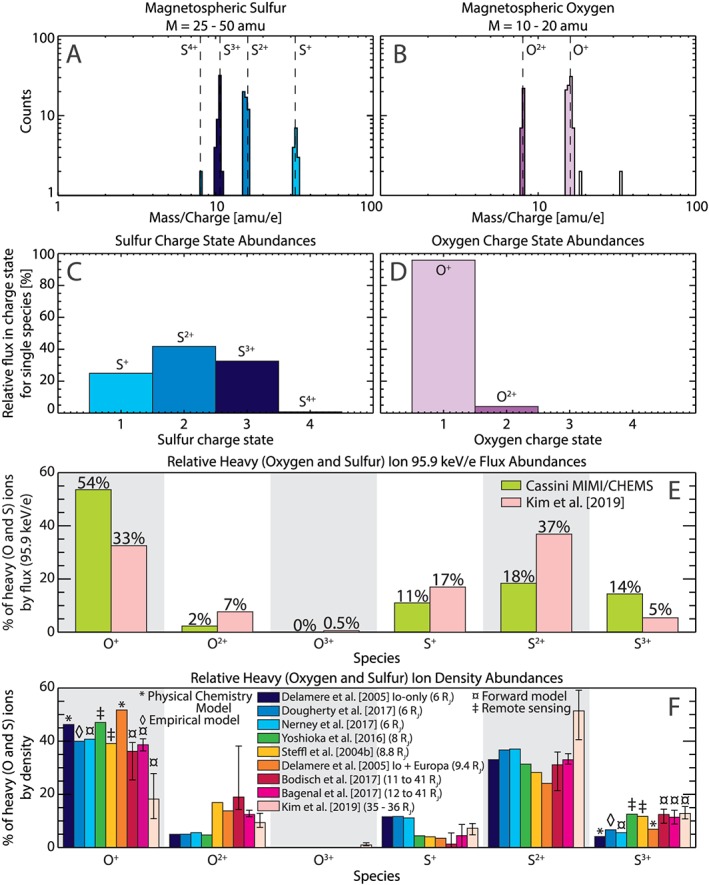
Histograms of counts binned by mass‐per‐charge for the mass range of (A) 25–50 amu and (B) 10–20 amu corresponding to sulfur and oxygen, respectively. Panels C and D illustrate the percentage of flux in each charge state for sulfur (oxygen). Panel E shows the relative flux abundance of 95.9 keV/*e* oxygen and sulfur from this study (green) and from Kim et al. (2019) (pink). Previously published relative densities are shown in Panel F. Error bars are included for surveys of multiple events to denote the maximum and minimum of the relative abundance within that study.

To compute the relative abundance of different charge state oxygen and sulfur, we isolate the magnetospheric interval PHA counts for the mass ranges of 10–20 and 25–50 amu. The different charge states of the sulfur and oxygen ions can be distinguished in Figures 4A and 4B, respectively. Since the peaks in counts for the different charge states are completely isolated from one another, we can compute the relative flux abundance of the charge states of sulfur ions (Figure 4C) and oxygen ions (Figure 4D). The highest ionization levels of 95.9 keV/*e* oxygen and sulfur that were appreciably measured (>1% of species by flux) were 2 (i.e., O^2+^) and 3 (i.e., S^3+^), respectively. However, the vast majority of oxygen ions by flux (96%) are O^+^, and the plurality of sulfur ions (42%) are S^2+^.

The relative flux abundance for the different charge states for oxygen plus sulfur ions is shown in Figure 4E. The green bars illustrate the relative abundance of the oxygen and sulfur charge states based on the findings of this paper, while the pink bars are the values from convected kappa distributions in Kim et al. (2019). These kappa distributions were fitted using a forward model, including instrument effects, of measurements taken by JADE‐I (McComas et al., 2013) onboard Juno within the plasma sheet at ~35 R_J_ and a local time of ~0400 hr. While the maximum observable energy of JADE‐I is 46.2 keV/*e*, the kappa distribution was used to estimate the relative fluxes at exactly 95.9 keV/*e* to compare to the same energy observed by Cassini. For more about these kappa fits, see Kim et al. (2019).

Examining the percentage of flux of the combined O and S group comprised of sulfur ions in Figure 4E, Cassini (green bars) observed less relative sulfur ion flux than seen at Juno (pink bars, with 43% at Cassini versus 59% at Juno) compared to relative oxygen ion flux (56% at Cassini versus 41% at Juno). However, among oxygen charge states observed by Cassini (Figure 4D), the relative flux of O^+^ increased (making up 96% of oxygen ions at Cassini versus 81% at Juno). Meanwhile, the relative composition of sulfur ion charge states observed by Cassini (Figure 4C) has an enhanced relative abundance of S^3+^ than at Juno (33% versus 9% of sulfur ions), while S^+^ and S^2+^ decreased in relative flux abundance for sulfur ions at Cassini compared to at Juno (25% versus 29% for S^+^ and 42% versus 62% for S^2+^).

Figure 4F illustrates the relative density abundance for each oxygen and sulfur ion charge state reported by nine previous studies (denoted by color) in the order of radial distance from Jupiter. The symbol above each bar in the first (O^+^) and last (S^3+^) charge state of the panel denotes the type of data used by that study. For example, both bars taken from Delamere et al. (2005) are physical chemistry model results with and without input from Europa, while Dougherty et al. (2017) is an empirical model based on Voyager PLS data. Meanwhile, both Yoshioka et al. (2017) and Steffl et al. (2004b) used remote sensing (Hisaki EUV and Cassini UVIS, respectively). Lastly, three studies of reanalysis from forward modeling of Voyager PLS observations are shown (Bagenal et al., 2017; Bodisch et al., 2017; Nerney et al., 2017), along with a forward model of Juno JADE‐I observations (Kim et al., 2019). While each of these studies makes its own assumptions (e.g., assuming the density of O^2+^ is 10% that of O^+^ in Yoshioka et al., 2017) and has its own uncertainties, caveats, spatial coverages, and energy ranges, the results are seen to qualitatively agree in regard to the relative order of abundance of the species. These comparisons will be further discussed in the following section.

## 4 Discussion

Using observations from MIMI/CHEMS during Cassini's excursion into the Jovian magnetosphere, we have reliable measurements of the relative abundance of all 95.9 keV/*e* oxygen and sulfur charge states. These observations provide a unique data point on this outstanding issue, being outer magnetospheric (~200 R_J_) suprathermal particles with unambiguous discrimination of mass and charge state, which can be compared to estimates from the inner to middle magnetosphere. However, because these observations comprise a single measurement in this region, temporal effects cannot fully be addressed.

Previous work using Voyager data found the S/O ratio decreases with radial distance in the inner to middle magnetosphere until leveling off near ~40 R_J_, reaching values between ~10% and 40% in the outer magnetosphere (Hamilton et al., 1981). This is consistent with the 43% relative sulfur flux observed by Cassini (~200 R_J_) being less than the 59% at Juno (~35 R_J_). This suggests S^n+^ is either lost faster or produced at a lower rate than O^n+^ until some point between ~35 R_J_ to 200 R_J_, when the S/O ratio levels off as seen in Hamilton et al. (1981).

Along with the 95.9 keV/*e* sulfur to oxygen ion ratios (S^n+^/O^n+^) being lower at Cassini than as estimated for Juno, the flux ratio of S^2+^ to all 16 amu/*e* ions (S^2+^/(O^+^+S^2+^)) also decreased (53% at 35 R_J_ compared to 26% at 200 R_J_). Understanding this difference may provide insight into the global relative abundance of S^2+^ to O^+^. The relative abundance of the other constituent charge states of oxygen and sulfur was also seen to be different between the Juno and Cassini locations.

These differences are possibly related to either the suprathermal ion populations reported by Kim et al. (2019) or that the charge state distribution is changing as the plasma is transported away from Jupiter. The convected kappa distributions begin to miss the suprathermal population of ions observed at higher energies, leading to a small discrepancy between the flux estimations at high energy and observations from JEDI (see discussion in Kim et al., 2019). Suprathermal ion population at higher energies (e.g., 95.9 keV/*e*) may have different relative abundances of both O^n+^ and S^n+^ along with different constituent charge states than the bulk population. For example, the known equilibrium charge state dependence on energy‐per‐charge in a sulfur‐H_2_ system for high energies (> 50 keV/*e*; Ozak et al., 2010). This may also explain why the high‐quality factor results from Clark et al. (2016), who used Galileo EPD data to infer the charge state distributions during ion injection events at 10–23 R_J_, found energetic (512–9920 keV) sulfur ions are largely S^2+^ and S^3+^. An energy dependence has also been observed for ion loss due to charge exchange, which would suggest higher charge states (for a given energy‐per‐charge) have longer lifetimes in the Jovian system, which could alter the charge state composition as it's transported outward (Lagg et al., 2003). Energetic oxygen neutral atoms, which may be the result of charge exchange with heated O^+^ ions in the inner magnetosphere, can also become reionized in the outer magnetosphere, adding to the relative energetic O^+^ content. Further work is required to better understand the charge state composition of this secondary energetic population seen in the Juno measurements and to determine if the changes in relative abundance of constituent charge states are a spatial effect and/or due to the energy of the population.

Comparing the results of the Delamere et al. (2005) physical chemistry model with and without the inclusion of the Europa source of particles (Figure 4F, deep navy versus orange) illustrates the importance of Europa for iogenic plasma as it is transported radially outward. Particularly for O^2+^, the inclusion of Europa as an additional source of oxygen appears to become important for the ion charge state composition beyond 9 R_J_. This effect may be slightly exaggerated in Delamere et al. (2005), as the Europa source was added by including 2 × 10^27^ O atoms/s at the orbit of Europa (based on Smyth & Marconi, 2006), which may be an overestimation of the Europa source, likely <5 × 10^26^ O atoms/s (see Bagenal et al., 2015). The relative abundances of the ion charge states don't seem to significantly change outside of the Europa torus orbit, likely due to the low density of neutrals. It should be stated, though, that we cannot directly compare the relative abundances of the densities with the flux ratios from CHEMS due to mass‐ and energy‐dependent terms in the density moment and the fact that the fluxes presented here (Figure 4E) are for one suprathermal energy and as such don't necessarily match the thermal bulk population dominating density calculations (Figure 4F).

Additionally, certain processes can lead to charge‐state‐dependent acceleration, which may contribute to observed differences in the Cassini and Juno observations. For example, wave‐particle interactions within Jovian plasmoids would further accelerate higher charge state species (Kronberg et al., 2019), while acceleration in reconnection exhausts is species dependent (e.g., Drake, Cassak, et al., 2009; Drake, Swisdak, et al., 2009; Vines et al., 2017). Future work is needed to better understand how these dynamical processes may shape the outer magnetospheric suprathermal ion composition.

Finally, comparing the flux measurements of the three CHEMS telescopes, most counts (67%) were observed in telescope 3, followed by 29% observed by telescope 2, and very few (4%) counts observed by telescope 1 (not shown). For this interval, this would be consistent with tail‐ward flow of the observed energetic plasma, since the average angles between the telescope boresights and the sun were 121°, 92°, and 62° for telescopes 1, 2, and 3, respectively. This is consistent with observed plasma flow paths along the outer flanks by the New Horizons spacecraft (McComas et al., 2007; McNutt et al., 2007).

## 5 Conclusions

In this letter, we investigated the relative flux abundance of 95.9 keV/*e* oxygen and sulfur ions at the outer (~200 R_J_) post‐dusk flank of the Jovian magnetosphere. This observation is compared with measurements of oxygen and sulfur ions closer to Jupiter to investigate changes of the charge state distributions in the outer magnetosphere. The main results are summarized as follows:

1. The highest charge state of appreciably measured sulfur in the outer magnetosphere was S^3+^, while the highest ionization state measured for oxygen was O^2+^, consistent with the inner magnetosphere.
2. The plurality of outer magnetospheric 95.9 keV/*e* sulfur is S^2+^ (42% of sulfur ions), followed in abundance by S^3+^ (33% of sulfur ions), and S^+^ (25% of sulfur ions). Compared to the Juno JADE‐I forward‐modeled 95.9 keV/*e* sulfur at ~35 R_J_ (see Kim et al., 2019), the Cassini observations at ~200 R_J_ observe a higher relative abundance of S^3+^ and lower relative abundance of S^+^ and S^2+^. This could suggest a charge‐dependent heating and/or energization effects in the outer magnetosphere or an energy dependence of the charge state abundance.
3. Most outer magnetospheric 95.9 keV/*e* oxygen is O^+^ (96% of oxygen ions), with the remaining oxygen ions being O^2+^ (4% of oxygen ions). This dominance of O^+^ is different from the inner and middle magnetosphere (e.g., Clark et al., 2016; Geiss et al., 1992; Kim et al., 2019). This could suggest either preferential acceleration of O^+^ to 95.9 keV/*e* or processes such as charge exchange and ionization and modify the relative abundance of oxygen charge states as the plasma is transported outward.
4. The flux of 95.9 keV/*e* O^+^ was ~3 times higher than for S^2+^. This is higher than estimated by Kim et al. (2019) near 35 R_J_ of 53% at Juno compared to 26% at Cassini, suggesting the relative abundance of O^+^ and S^2+^ (m/q = 16 amu/*e*) particles changes as the plasma is transported to the outer magnetosphere.
5. Total 95.9 keV/*e* oxygen ion flux (O^+^ + O^2+^) was 56% of the combined 95.9 keV/*e* oxygen and sulfur ion flux making the O^n+^ to S^n+^ ratio ~1.3. This agrees well with early results at higher energies from Voyager 1 and 2 in the outer magnetosphere (Hamilton et al., 1981), comparable to neutral cloud models (matching ultraviolet emissions) at thermal energies near 9 R_J_ (Delamere et al., 2005), but is greater than the Juno kappa fits from Kim et al. (2019) at ~35 R_J_.
